# Case report: A case of anti-glycine receptor encephalomyelitis triggered by post-transplant or COVID-19 infection?

**DOI:** 10.3389/fneur.2024.1356691

**Published:** 2024-04-18

**Authors:** Zhengxue Zhang, Xiang Zhang, Mingming Dai, Yingying Wu, Yong You

**Affiliations:** ^1^Department of Neurology, The Second Affiliated Hospital of Hainan Medical University, Haikou, China; ^2^Department of Neurology, Huashan Hospital, Fudan University, Shanghai, China; ^3^International Center for Aging and Cancer (ICAC), Hainan Medical University, Haikou, China; ^4^Key Laboratory of Brain Science Research & Transformation in Tropical Environment of Hainan Province, Haikou, China

**Keywords:** post-transplant, COVID-19 infection, anti-glycine receptor encephalomyelitis, magnetic resonance imaging, treatment outcome

## Abstract

Even though long-term immunosuppressant drugs (ISD) are employed to inhibit immune system activity, enhancing graft functionality and patient survival in solid organ transplantation (SOT), these transplants often lead to immune complications, with post-transplant autoimmune diseases of the central nervous system (CNS) being uncommon. Here, we detail the case of a 66-year-old woman who underwent a renal transplantation 8 months prior, who was admitted with subacute onset of encephalomyelitis, accompanied by headaches, paraplegia, weakness, vomiting, and abdominal pain, with a positive COVID-19 nasopharyngeal swab test 1 month before admission. MRI scans of the brain revealed multiple lesions in the white matter of the bilateral deep frontal lobe, the left temporal lobe and insula lobe. Additionally, there were multiple short segment lesions in the spinal cord and subdural hematoma at T1, T6-T7 posterior. The serum revealed a positive result for GlyR-IgG. Following the administration of corticosteroid and intravenous immunoglobulin, there was a significant improvement in the patient’s symptoms within 2 weeks, and her brain MRI showed a reduction in the lesion. Despite its rarity, we believe this to be the inaugural documentation of anti-GlyR encephalomyelitis occurring during renal transplantation. A full panel of antibodies for autoimmune encephalomyelitis is the key leading to the diagnosis.

## Introduction

Anti-glycine receptor (GlyR) encephalomyelitis was first described by Hutchinson in 2008 (1). Typical clinical symptoms encompass progressive encephalomyelitis with rigidity and myoclonus (PERM), stiff person syndrome (SPS), limbic encephalitis, epileptic encephalopathy, and demyelinating optic neuropathies (2). Brain MRI scans reveal pathological conditions in 27.8% of patients, with spinal cord lesions in 21.7% exhibiting extensive lesions over time (2).

Approximately one-third of the patients are likely to experience neurologic disorders including seizures, cerebrovascular events, and opportunistic infections within 1 month following an SOT operation (3). Earlier, several case studies have reported post-transplant neuro-immunological diseases, such as anti-N-methyl-D-aspartate receptor (NMDAR) encephalomyelitis (4, 5) and anti-alpha-amino-3-hydroxy-5-methyl-4-isoxazolepropionic acid receptor (AMPAR) encephalomyelitis (6, 7).

To the best of our knowledge, this is the first case study on the development of anti-GlyR encephalomyelitis in a patient infected with COVID-19 who underwent a successful renal transplantation 8 months prior.

## Case description

A 66-year-old female patient came to our hospital with headaches, paraplegia, fatigue, incontinence, and abdominal pain for 5 days, and soon developed severe focal impaired awareness seizures. 8 months ago, she had received a renal transplantation for end-stage renal disease secondary to chronic glomerulonephritis. Maintenance immunosuppression at that time included methylprednisolone (4 mg), tacrolimus (2 mg), and mycophenolate mofetil (MMF, 750 mg) to prevent a graft-versus-host response. A month prior to her admission, she was in close contact with a COVID-19 infection patient, then developed a 38.0 degrees Celsius and tested positive for a COVID-19 nasopharyngeal swab, known for the first COVID-19 infection, without vaccination after renal transplant.

**Figure 1:**
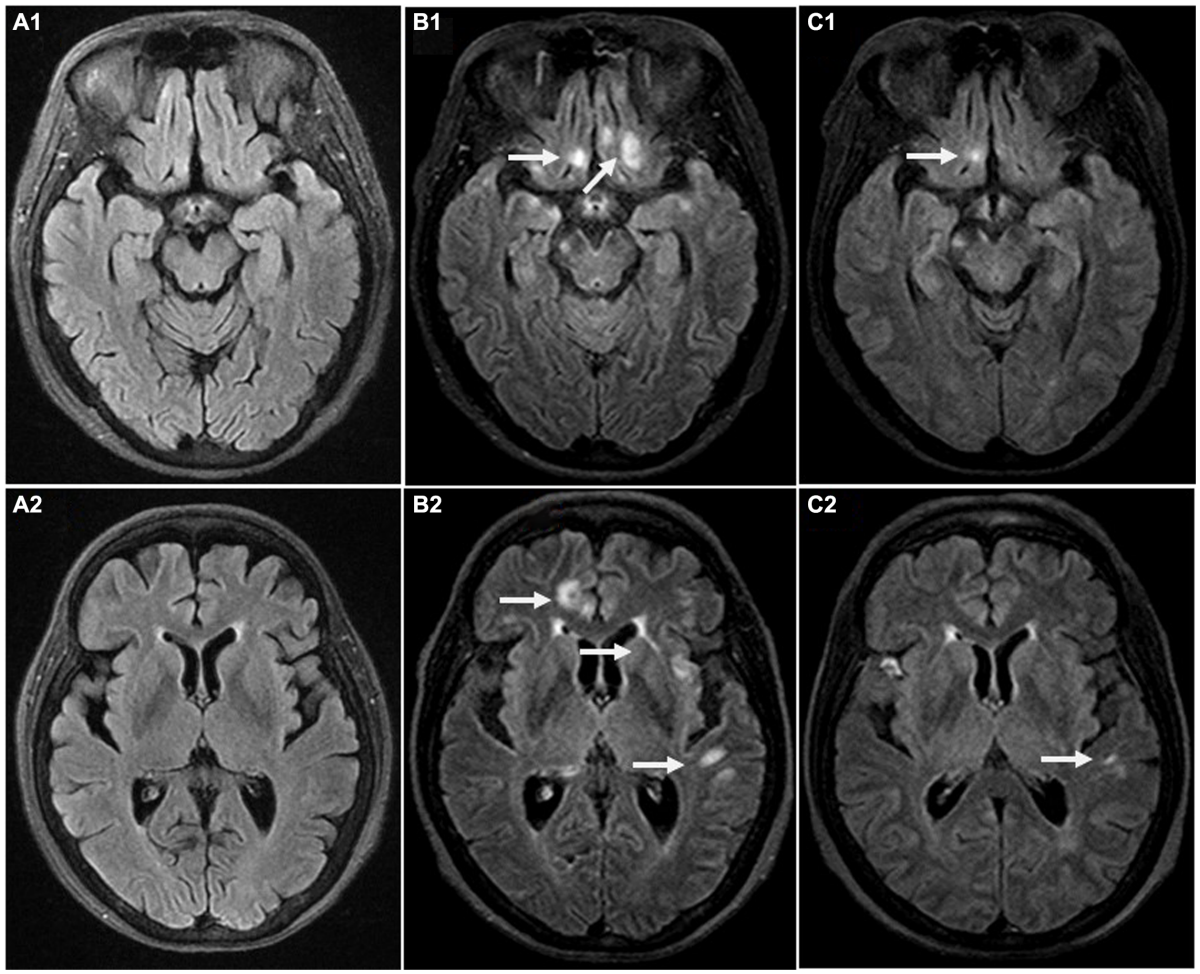
Brain magnetic resonance imaging (MRI) at the time of pre-onset (**A1,A2**, Oct 4, 2022 date), ongoing disease (**B1,B2**, Feb 3, 2023 date) and post-immunotherapy (**C1,C2**, Feb 22, 2023 date). T2-weighted fluid-attenuated inversion recovery (FLAIR) sequence in the axial orientation shows normal brain parenchyma **(A1,A2)**. multiple patchy long T2 signals in the white matter of the bilateral deep frontal lobe **(B1)**, and in the left temporal lobe, insula lobe and right deep frontal lobe **(B2)**. Repeat MRI shows marked reduction in the area of the frontal lobe and the left temporal lobe after immunotherapy **(C1,C2)**. Arrows indicate focal lesions.

**Figure 2:**
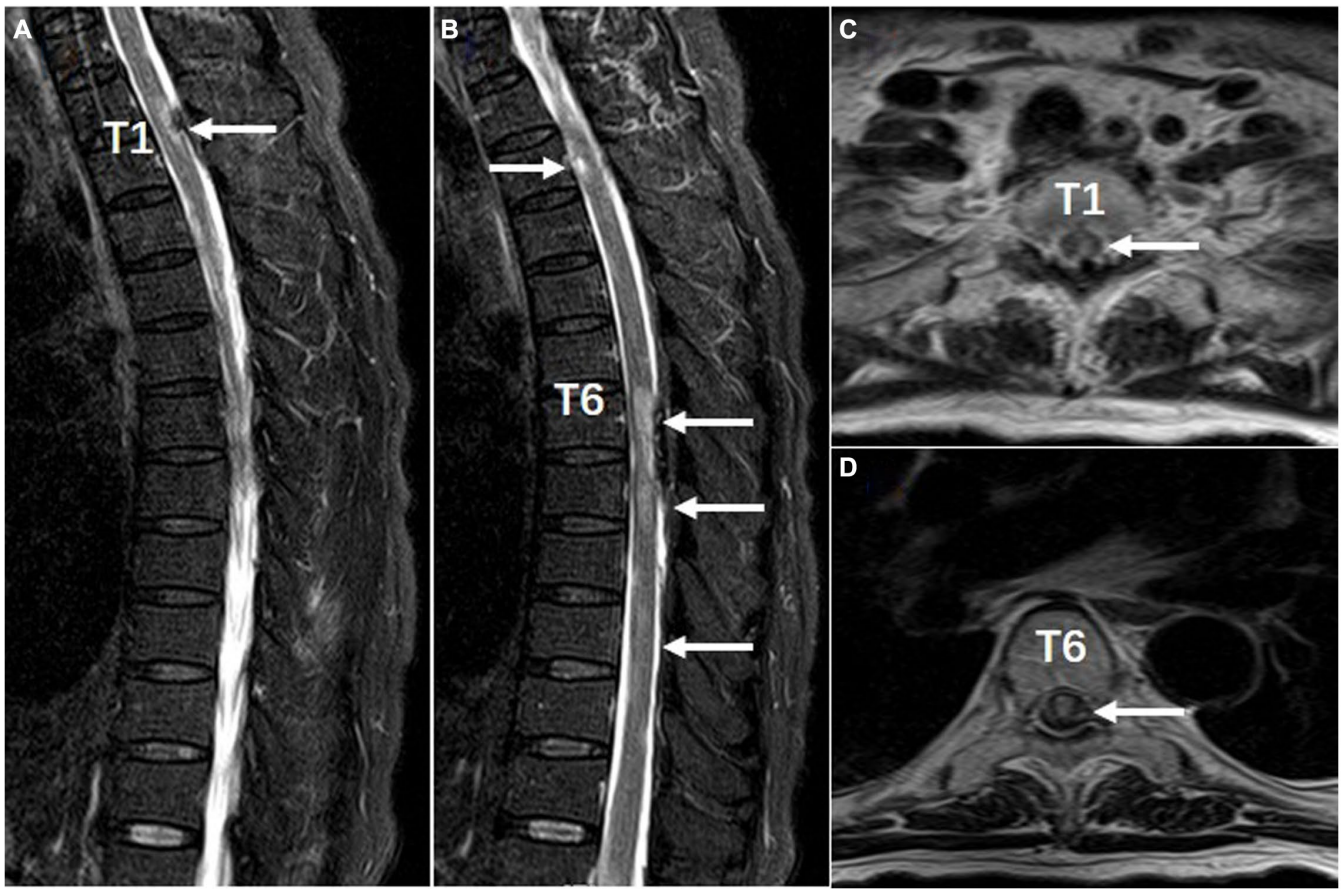
Spinal MRI performed with T2-weighted sagittal **(A,B)** and axial **(C,D)** views (Feb 8, 2023 date). There are multiple short segment lesions in the spinal cord and subdural hematoma at T1, T6-T7 posterior. Arrows indicate focal lesions.

After admission, the patient underwent repeated COVID-19 nasopharyngeal swab tests, which came back negative and her temperature was normal. She had paraplegia, no deep tendon reflex, and loss of sensation below T1 level. The Expanded Disability Status Scale (EDSS) scored 8.5. Chest CT scan showed diffuse interstitial inflammation in both lungs. Brain MRI displayed multifocal lesions in subcortical areas (Figures 1B1,B2) that were not presented 4 months ago (Figures 1A1,A2). She had hyponatremia (Na, 128.2 mmol/L) and hypochloremia (Cl, 93.6 mmol/L). The serum creatinine level was 109 μmol/L. Routine laboratory studies including routine blood tests, C-reactive protein, coagulation tests, and liver function tests were all unremarkable. Serum procalcitonin (PCT) was 0.22 ng/mL (<0.1 ng/mL, reference) and IL-6 was 9.88 pg./mL (0–6.61 pg./mL, reference). GlyR antibodies were shown to be positive (titer 1:100) in serum upon suspicion of autoimmune disease of the CNS. The antibodies linked to autoimmune encephalitis were found by a cell-based assay (Guangdong Jinyu Inspection Company, Guangzhou, China). An MRI scan of the spinal cord was then performed, revealing multiple swollen short lesions in the thoracic cord accompanied by a subdural hematoma (Figures 2A–D). Radiological detection ruled out possible underlying neoplasms. Unfortunately, her family refused to have a lumbar puncture or an electroencephalogram (EEG) examination.

**Figure 3:**
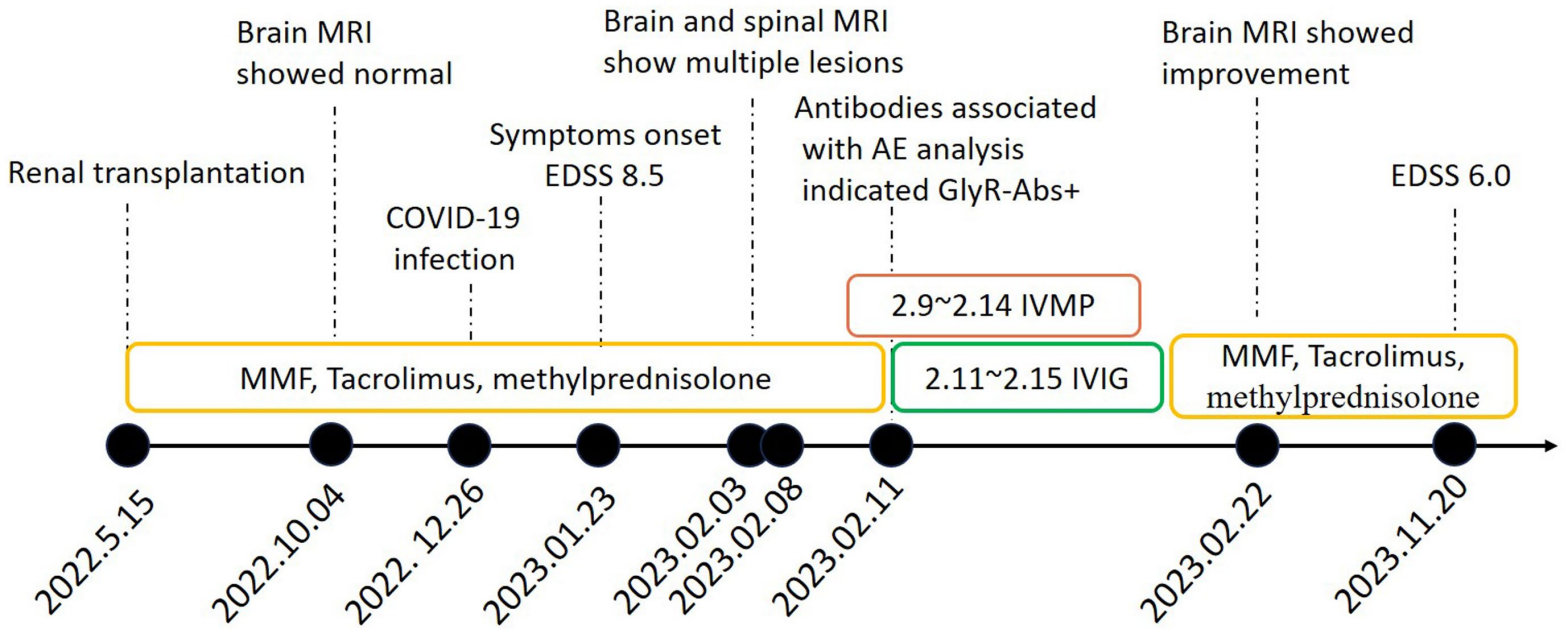
Timeline of clinical events, diagnostic work-up and treatment during the disease course. MRI magnetic resonance imaging; MMF mycophenolate mofetil; EDSS Expanded Disability Status Scale; AE autoimmune encephalitis; GlyR-Abs glycine receptor antibodies; IVMP Intravenous methylprednisolone; IVIG intravenous immunoglobulins.

Valproate successfully controlled seizures. She was given intravenous methylprednisolone (IVMP, 500 mg/d for 6 days) and intravenous immunoglobulin (IVIG, 0.4 g/kg d for 5 days). Her symptoms eased noticeably and she restored to her normal mental status. 19 days after the treatment, an MRI review of her head showed that most lesions had decreased or disappeared in previous examinations (Figures 1C1,C2). Then she was discharged with methylprednisolone (4 mg), Tacrolimus (2 mg), and MMF 750 mg orally taken every day. After 9 months of follow-up, her condition was stable and her EDSS dropped to 6.0. We also organized patient clinical events, diagnostic work-up, and treatment options as a timeline (Figure 3).

## Discussion and conclusions

GlyRs are ligand-gated chloride channels, that facilitate inhibitory signaling, presenting mainly in the brainstem and spinal cord (8). GlyR expression is also observed in GABAergic synapses within certain hippocampal interneurons and pyramidal neurons (9). The presence of GlyR autoantibody hinders GlyR activity and invariably leads to motor dysfunction (10) and pain (11). Despite numerous previous studies linking GlyR antibodies to SPS/PERM (1), the range of diseases related to GlyR antibodies varies greatly. Reports also indicate conditions such as muscle stiffness and rigidity, myoclonus, eye movement disorders, bulbar issues, heightened startle reflex, painful spasms, autonomic disturbances, seizures, and cognitive impairment (2). In this case, the patient presented symptoms like headache, weakness in limbs, loss of bladder control, and stomach pain, yet lacked rigidity and myoclonus. MRI detection indicated multiple lesions in the subcortical white matter and spinal cord, resembling acute disseminated encephalomyelitis (ADEM). The presence of these atypical symptoms complicates linking the disease to GlyR antibodies. However, a COVID-19 infection could potentially explain the subdural hematoma. Possible mechanisms include the virus invade and directly damage cerebral vessels, an inflammatory storm mediate vascular remodeling, and autoregulation disruption (12, 13).

This case is unique since it happened to the patient following a renal transplantation, and the patient had previously contracted a COVID-19 infection. Determining the primary source of GlyR antibody production was made more challenging by the presence of these intricate circumstances.

Since there are few reports of encephalitis linked to transplants, more research is needed to determine the precise processes. According to earlier findings, the majority of post-transplant autoimmune encephalitis develops more than a year after transplantation, with the time interval between transplantation and the onset of the condition ranging from 1 month to 14 years (4–7, 14). Our patient’s disease began 8 months post-renal transplantation, showing no evidence of chronic allograft rejection response. Moreover, factors such as infections, neoplasms, and immune checkpoint inhibitors could play a crucial role in initiating autoimmune reactions. Pathologic mechanisms include molecular mimicry, bystander activation of T lymphocytes, transient immunosuppression, and inflammation (15). Molecular mimicry is the primary suspect implicated in the pathophysiology of autoimmunity during COVID-19 infection (16). Furthermore, COVID-19 may result in autoimmune encephalitis (AE), which may manifest as limbic encephalitis, anti-NMDA receptor encephalitis, steroid-responsive encephalitis, AE presenting as new-onset refractory status epilepticus (NORSE), and unknown type of AE (17). A solitary case report of GlyR antibody-associated autoimmunity was described during the SARS-CoV-2 infection (18), akin to the situation with our patient who experienced a COVID-19 infection a month earlier.

Despite extended use of steroids and immune-modulators post-renal transplantation in our case, GlyR autoimmunity still develop. We suggest that the decline and dysregulation of immune function related to ISD could have increased susceptibility to COVID-19 (19), leading to GlyR molecular mimicry in renal transplant patients, resulting in symptoms resembling ADEM. Due to the rarity of such situations, more in-depth explanations and research are needed. Fortunately, there was an improvement in this patient’s clinical symptoms and radiological signs, suggesting a positive response to immunotherapy.

## Data availability statement

The datasets presented in this article are not readily available because of ethical and privacy restrictions. Requests to access the datasets should be directed to the corresponding authors.

## Ethics statement

The studies involving humans were approved the Ethics Committee of the Second Affiliated Hospital of Hainan Medical University (Approval number: LW2023101). The studies were conducted in accordance with the local legislation and institutional requirements. The participants provided their written informed consent to participate in this study. Written informed consent was obtained from the individual(s) for the publication of any potentially identifiable images or data included in this article.

## Author contributions

ZZ: Data curation, Visualization, Writing – original draft, Writing – review & editing. XZ: Formal analysis, Writing – review & editing, Conceptualization. MD: Formal analysis, Writing – review & editing, Methodology, Resources. YW: Methodology, Writing – review & editing, Validation. YY: Validation, Writing – review & editing, Formal analysis, Funding acquisition, Supervision.
